# A Herbal Formula HT051, a Combination of* Pueraria lobata* and* Rehmannia glutinosa*, Prevents Postmenopausal Obesity in Ovariectomized Rats

**DOI:** 10.1155/2017/8641535

**Published:** 2017-12-26

**Authors:** Yoon Hee Lee, Bora Jin, Sunghyun Lee, Jin-Young Oh, Jungbin Song, Donghun Lee, Young-Sik Kim, Hocheol Kim

**Affiliations:** ^1^Korea Institute of Science and Technology for Eastern Medicine (KISTEM), NeuMed Inc., Seoul 130-701, Republic of Korea; ^2^Department of Herbal Pharmacology, College of Korean Medicine, Kyung Hee University, Seoul 130-701, Republic of Korea

## Abstract

Menopause is strongly associated with an increased risk of metabolic dysfunctions due to the decline in estrogen. Here, we hypothesized that dietary HT051, containing the roots of* Pueraria lobata* and* Rehmannia glutinosa*, has beneficial effects on ovariectomized (OVX) rats by regulating lipid metabolism. Forty-eight female Sprague-Dawley rats were randomly divided into 4 groups: sham-operated (Sham), OVX, OVX with low-dose HT051 supplementation, and OVX with high-dose HT051 supplementation. The rats were fed with a modified AIN-93G diet or an HT051-containing modified AIN-93G diet for 8 weeks. Body weight, fat mass, and serum levels of total cholesterol, triglyceride, glucose, alanine transaminase, and aspartate transaminase decreased in HT051-fed OVX rats. Dietary HT051 supplementation significantly decreased the mRNA expression of lipogenesis-related genes, including sterol regulatory element-binding protein 1c and fatty acid synthase, and increased the mRNA expression of *β*-oxidation-related genes, including peroxisome proliferator-activated receptor and carnitine palmitoyl transferase 1 in the liver of OVX rats. Moreover, the expression of genes involved in adipogenesis and inflammation was significantly lower in the adipose tissue of OVX rats fed with HT051 than in the OVX group. These findings suggest that HT051 may be a potential natural alternative for the management of postmenopausal metabolic dysfunctions.

## 1. Introduction

Menopause is a biological stage in a woman's life when menstrual cycles cease resulting from the loss of ovarian function and estrogen deprivation [1, 2]. Although some women undergo early menopause due to the surgical removal of ovaries, or through radiation or chemotherapy, generally menopause is an event that occurs as a result of ageing, and most women experience menopause within the range of 40 and 58 years of age [3]. Postmenopause is defined as the time of the final menstrual period, followed by one year after the last period.

Postmenopause is strongly associated with an increased risk of obesity owing to estrogen level depletion [4]. Estrogen regulates insulin sensitivity of the pancreas, liver, and skeletal muscle and suppresses fat distribution, differentiation, and fibrosis of white adipose tissue (WAT) and induces thermogenesis of brown adipose tissue (BAT), thus impacting lipid metabolism [5]. Thus, estrogen deprivation strongly affects adipocyte differentiation and brings about a redistribution of fat, leading to increased visceral fat stores [6]. Postmenopausal women have greater amounts of the visceral body fat compared with premenopausal women. Increased visceral fat is strongly associated with insulin resistance and inflammation, which are key factors for developing metabolic dysfunctions, including type 2 diabetes mellitus, cardiovascular disease, and nonalcohol fatty liver disease (NAFLD) [6, 7]. Moreover, higher abdominal adiposity, particularly subcutaneous adiposity, is a key risk factor of vasomotor symptoms (VMS) for early postmenopause [8]. VMS are the most common initial menopause symptom, often called “hot flashes,” and are characterized by a sudden intense heat, often to the face, neck, and chest, and spread throughout the body. VMS disturb the quality of life by affecting sleep, mood, and cognitive function [9]. Therefore, the management and prevention of postmenopausal obesity are extremely important for maintaining health and improving quality of life in the postmenopausal populace.

Hormone replacement therapy (HRT) is the strategy currently used for preventing and treating the symptoms of postmenopause [10]. Ironically, long term HRT has been associated with increased risk of undesired side effects including headache, fluid retention, swollen breasts, breast cancer, endometrial cancer, venous thromboembolism, and cardiovascular disease [11, 12]. In other words, there are no treatments that can be used safely in the long term in the management of postmenopausal syndromes. Thus, it is necessary to develop a new drug of natural or synthetic origin, with minimal side effects.

Many natural products have been used in clinical practice to enhance the health of postmenopausal women. For improving postmenopausal syndrome, we selected two herbs, the roots of* Pueraria montana* var.* lobata* (Willd.) Sanjappa & Pradeep (Leguminosae) and* Rehmannia glutinosa* (Gaertn.) DC. (Plantaginaceae), and designated the resulting formula as HT051.* P. lobata*, also known as Galgeun, has been traditionally used for reducing dry mouth, promoting circulation, increasing the blood flow, and treating alcoholism, cardiovascular disease, and type 2 diabetes mellitus [13]. Recent studies have demonstrated that* P. lobata* improves glucose tolerance in* ob/ob* mice, reduces body weight gain and serum lipid levels in obese mice, inhibits skeletal muscle atrophy in obese mice, and prevents bone loss in ovariectomized (OVX) mice [14, 15].* P. lobata* contains a high content of isoflavonoids, especially puerarin, daidzin, daidzein, and genistein [16]. Isoflavonoids are a type of phytoestrogen that can produce an estrogen-like effect, used as a natural alternative to HRT to improve menopausal symptoms [17].* R. glutinosa*, also known as Jihwang, has been widely used as traditional Chinese and Korean medicine for improving the circulatory system, cardiovascular system, nervous system, immune system, and bone metabolism [18].

A combination of* P. lobata* and* R. glutinosa* has been reported to prevent osteoporosis, reduce body weight, and induce fat oxidation of skeletal muscle in an OVX animal model [19–21]. A recent study has demonstrated that the combination of* P. lobata* and* R. glutinosa* extracts shows an antiosteoporosis effect by inhibiting the expression of receptor activator of nuclear factor kappa-B ligand (RANKL), which induces osteoclast activation and bone resorption in the OVX animal model [19]. Supplementation of a mixture of* P. lobata* and* R. glutinosa* increased fat oxidation through the upregulation of plasma membrane-bound fatty acid binding protein (FABPpm) in skeletal muscle [20]. Moreover, a combination of* P. lobata* and* R. glutinosa* extracts decreased body weight, retroperitoneal fat, and perirenal fat [21]. However, the mechanism underlying the antiobesity activity of a combination of* P. lobata* and* R. glutinosa* extracts has not been studied yet. The aim of this study was to investigate the antiobesity effect of HT051, a* P. lobata and R. glutinosa* mixture, and its mechanisms in OVX rats. In the present study, we examined body weight, WAT weight, serum lipid levels, and the expression of genes involved in adipogenesis, lipogenesis, *β*-oxidation, and inflammation in the liver and WAT of OVX rats to elucidate potential underlying mechanisms of HT051.

## 2. Materials and Methods

### 2.1. Plant Material

The dried roots of* P. lobata* and* R. glutinosa* were purchased from Dongkyung Co. (Seoul, Republic of Korea). They were identified by Professor Dr. Hocheol Kim, and the voucher specimens (#HP028 and #HP130) were deposited at the Department of Herbal Pharmacology, College of Korean Medicine, Kyung Hee University (Seoul, Republic of Korea).

### 2.2. Sample Preparation and High Performance Liquid Chromatography (HPLC) Analysis

**Figure 1:**
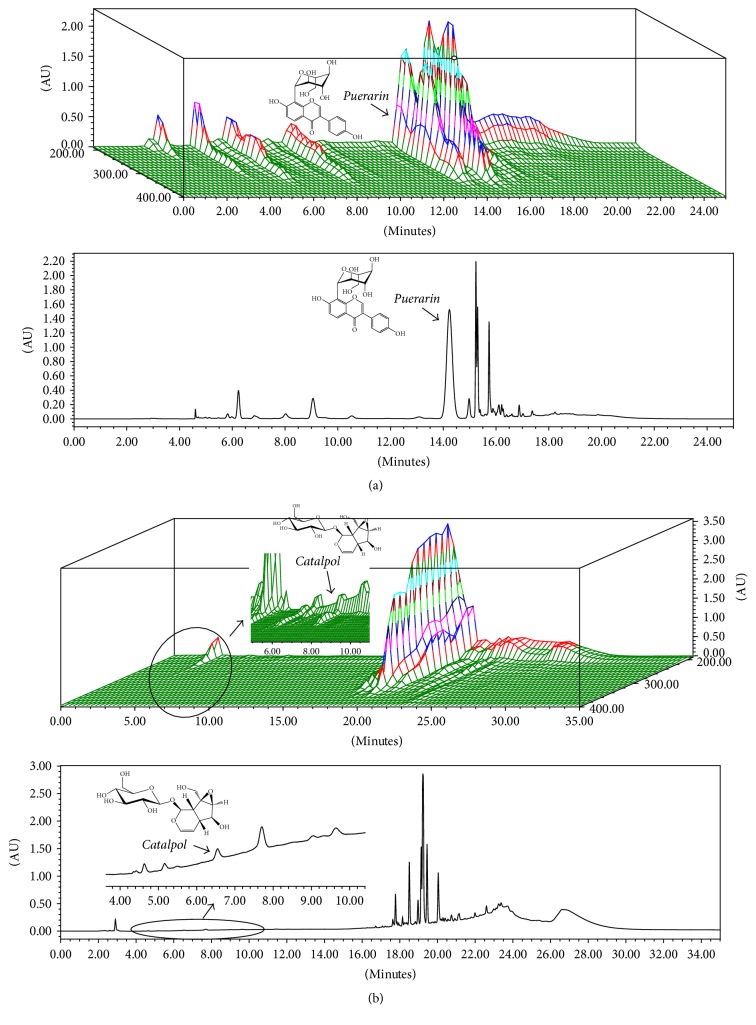
3D and 2D HPLC chromatograms of HT051, a blend of two herbal extracts: (a) puerarin of* P. lobata* root; (b) catalpol of* R. glutinosa* root.

The dried roots of* P. lobata* and* R. glutinosa* were extracted separately with water for 4 h twice at 100°C in a reflux apparatus. The extracts were filtered and concentrated under reduced pressure, and samples were spray-dried with 20% dextrin for* R. glutinosa *and no dextrin for* P. lobata*. The extract yield of* R. glutinosa* was 45.6% and the yield for* P. lobata* was 29.6%. For the preparation of HT051,* P. lobata* and* R. glutinosa* extracts were mixed at a ratio of 2.4 : 1. The quantitative authentication of HT051 was performed by a HPLC analysis system equipped with a Waters 1525 pump, a 2707 autosampler, and a 2998 PDA detector (Waters, Milford, MA, USA). The chromatic separation was achieved at 40°C on Waters Sunfire™ C18 (250 mm × 4.6 mm i.d., 5 *μ*m particle size) column. The gradient program to analyze puerarin, which is a representative component of* P. lobata,* was as follows: 0–10 min, 12-12%; 10–15 min, 12–65%; 15–17 min, 65-65%; 17-18 min, 65–12%; 18–25 min, 12-12% solvent B. The gradient program to analyze catalpol, a representative component of* R. glutinosa*, was as follows: 0–10 min, 2-2%; 10–20 min, 2–45%; 20–23 min, 45-45%; 23–25 min, 45–2%; 25–35 min, 2-2% solvent B. The flow rate was 1 mL/min and the injection volume was 10 *μ*L. The puerarin and catalpol were monitored at 254 nm and 205 nm, respectively. Each extract was analyzed in triplicate. The content of puerarin and catalpol was calculated for standardization. In HT051, the content of puerarin was 65.27 ± 1.31 mg/g and catalpol was 3.21 ± 0.01 mg/g. A 3D chromatogram of HT051 is shown in Figure 1.

### 2.3. Animals and Treatments

Female Sprague-Dawley (SD) rats, 8 weeks old (170–190 g), were purchased from Samtako (Osan, Republic of Korea). The animals were housed in polycarbonate cages (3 rats/cage) under controlled temperature (23 ± 2°C), relative humidity (55–60%), and lighting conditions (lights on from 07:00 hours to 19:00 hours) with food and water made available ad libitum. Animal experiments were reviewed and approved by the Institutional Animal Care and Use Committee of Korea Institute of Science and Technology for Eastern Medicine (KISTEM) (project number: KISTEM-IACUC-2016-002; date of approval: 29 April 2016), and the animals were cared for according to the Guidelines for the Institutional Animal Care and Use Committee of Korea Institute of Science and Technology for KISTEM.

After acclimatization for 1 week, 9-week-old female SD rats were anesthetized with 5% isoflurane, and ovaries were removed bilaterally. The rats were divided into the four following treatment groups (*n* = 12 per group): Group 1, Sham group that had sham surgery and received a modified AIN-93G diet in which soybean oil was replaced with corn oil (Saeronbio Inc., Uiwang, Republic of Korea); Group 2, CON group that had ovariectomy and received a modified AIN-93G diet; Group 3, 1.0% HT051 group that had ovariectomy and received a modified AIN-93G diet containing 1.0% HT051; Group 4, 0.3% HT051 group that had ovariectomy and received a modified AIN-93G diet containing 0.3% HT051. The composition of the diets is shown in Table 1. All groups were treated for eight weeks. During the experimental period, body weight and food intake were determined weekly.

At the end of the treatment period, the rats were fasted for 12 h, and blood was collected via the abdominal aorta. The rats were anesthetized with N_2_O/O_2_ gas and isoflurane. The serum samples were prepared by centrifugation of the collected blood samples (1,300*g* for 10 min at 4°C) and then stored at −80°C for biochemical determinations. Liver, spleen, WAT, and uterus tissue were dissected, washed with saline solution, weighed, and stored at −80°C until further analysis.

### 2.4. Biochemical Serum Analysis

Serum ALT (98-24010-US), AST (98-24016-US), TC (98-24005-US), glucose (98-24009-US), and TG (98-24019-US) concentrations were measured using an autoanalyzer (IDEXX VetTest® Chemistry Analyzer, IDEXX Laboratories, Inc., Westbrook, ME, USA) according to the manufacturer's instructions. The VetTest apparatus requires 70 ul volumes of serum for all parameters. The different biochemical tests are available as dry slides that include all necessary reagents. Serum estradiol level was determined using Enzyme-Linked Immunosorbent Assay (ELISA) kit (ab108667, Abcam, Cambridge, UK) according to the manufacturer's instructions.

### 2.5. Real-Time Quantitative PCR Analysis

Total RNA from liver and WAT were extracted using Qiazol reagent (Invitrogen Technologies, Waltham, MA, USA). According to the manufacturer's instruction, the total RNA concentration and 260/280 nm ratio were evaluated using an Epoch 2 Microplate Spectrophotometer (BioTek Instruments, Inc., Winooski, VT, USA). Single strand RNA samples were converted to cDNA using the High Capacity cDNA Reverse Transcription Kit (Applied Biosystems, Foster City, CA, USA). The cDNA was performed using the Step-One-Plus RT-PCR System (Applied Biosystems, USA) as follows: after 10 min at 95°C, 40 cycles of 15 s at 95°C and 60 s at 60°C, followed by the melting curve for 15 s at 95°C for 15 s, a gradual decrease to 60°C in the last 60 s, and then a gradual increase to 95°C for the last 15 s. Primers were designed using nucleotide sequence and synthesized by Bioneer (Daejeon, Republic of Korea). The relative gene expression was normalized using the housekeeping gene (GAPDH). The sequences of the primers used in this study are listed in Table 2. The relative fold change of gene expression was calculated using the Delta-Delta method.

### 2.6. Statistical Analysis

Statistical analysis was performed using SAS 9.3 (SAS Institute Inc., Cary, NC, USA). All data were presented as the mean ± standard deviation (SD). The effects of different treatments were compared by one-way ANOVA test, followed by the post hoc Duncan's test for multiple comparisons. *p* < 0.05 was considered statistically significant.

## 3. Results

### 3.1. Weekly Body Weight and Weight Gain

**Figure 2:**
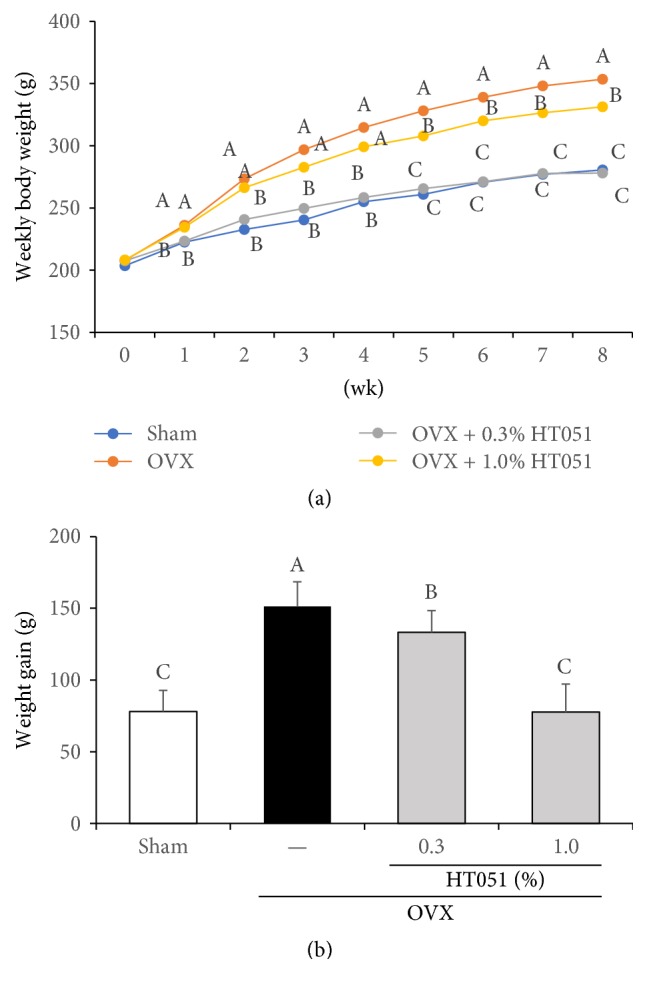
Effects of HT051 on weekly body weight (a) and weight gain (b) in ovariectomized rats. Each value is the mean ± SD (*n* = 12 per group). Data were analyzed by one-way analysis of variance (ANOVA) followed by Duncan's multiple range test. Values not sharing the same letters are significantly different among the groups at *p* < 0.05.

The values of weekly body weight, body weight gain, and food intake in OVX rats measured during the 8-week administration of HT051 are shown in Figure 2 and Table 3. The weekly body weight showed a significant difference between Sham and OVX groups from the first week through the entire experimental period (*p* = 0.0073, Figure 2(a)). 1.0% HT051 supplementation also significantly reduced weekly body weight from week 1 compared to that in the OVX group until the end of the experiment. Furthermore, the weekly body weight of the 0.3% HT051 group was significantly different from week 5 compared to that of the OVX group until the end of the experimental period (*p* < 0.0001). The body weight gain showed a significant difference between the OVX group and the other groups (*p* < 0.0001, Figure 2(b)).

### 3.2. Organ Weights

Table 4 shows the weight of the liver, spleen, and WAT (total WAT, mesenteric, abdominal, gonadal, and perirenal fat). As shown in Table 4, the weight of the liver and total WAT of the OVX group was significantly higher than those of the Sham group after the feeding period. HT051 supplementation decreased the weight of the liver and total WAT compared to that in the OVX group (*p* = 0.0134 and *p* < 0.0001, resp.). A significant difference was found in the total fat weight including mesenteric, abdominal, gonadal, and perirenal fat between the OVX group and HT051 groups (*p* < 0.0001, *p* < 0.0001, *p* < 0.0001, and *p* < 0.0001, resp.). In the HT051-supplemented groups, all WAT weights were decreased dose-dependently compared to the OVX group. There was no difference in the weight of the spleen among groups.

### 3.3. Uterus Weight and 17*β*-Estradiol Contents in Serum

**Figure 3:**
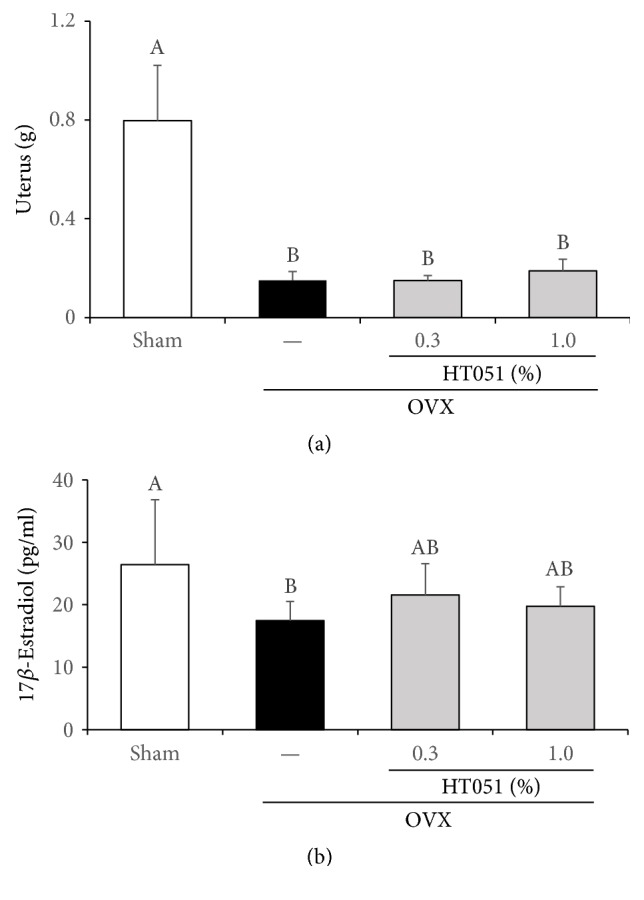
Effects of HT051 on uterus weight (a) and serum 17*β*-estradiol level (b) in ovariectomized rats. Each value is the mean ± SD (*n* = 12 per group). Data were analyzed by one-way analysis of variance (ANOVA) followed by Duncan's multiple range test. Values not sharing the same letters are significantly different among the groups at *p* < 0.05.

Ovariectomy resulted in a significant reduction in uterus weight in the ovariectomized rats, and there was no uterus hypertrophy in HT051-supplemented groups (*p* < 0.0001, Figure 3(a)). Serum 17*β*-estradiol levels were significantly lower in OVX rats compared to that in the Sham group (*p* = 0.0064). However, there was no significant difference in 17*β*-estradiol level between the OVX group and two OVX + HT051 groups (Figure 3(b)).

### 3.4. Biochemical Analysis of Serum

**Figure 4:**
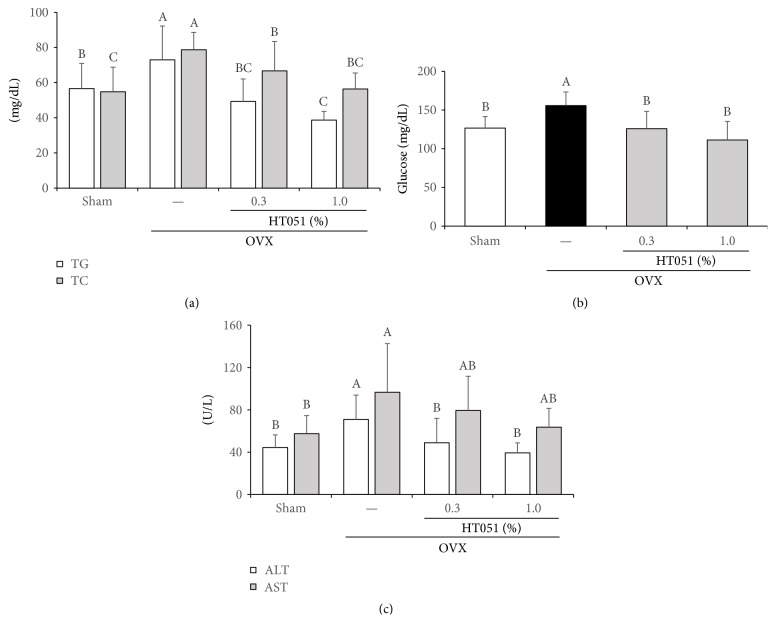
Effects of HT051 on serum lipid (a), fasting glucose (b), and ALT and AST (c) in ovariectomized rats. Each value is the mean ± SD (*n* = 12 per group). Data were analyzed by one-way analysis of variance (ANOVA) followed by Duncan's multiple range test. Values not sharing the same letters are significantly different among the groups at *p* < 0.05.

Serum levels of triglyceride (TG) and total cholesterol (TC) were significantly higher in the OVX group compared with the Sham group (*p* < 0.0001 and *p* = 0.0001, resp.). Blood lipid levels were significantly lower in two OVX + HT051 groups (Figure 4(a)). The level of fasting glucose in the OVX group was also significantly higher than that in the Sham group. As expected, fasting glucose level was significantly lower in two OVX + HT051 groups that that in the OVX group after 8 weeks (*p* < 0.0001, Figure 4(b)). In addition, alanine transaminase (ALT) levels were significantly lower in two OVX + HT051 groups compared to that in the OVX group (*p* < 0.0001). Serum aspartate transaminase (AST) levels of two OVX + HT051 groups were lower than that of the OVX group, but there was no significant difference (Figure 4(c)).

### 3.5. Lipogenesis- and *β*-Oxidation-Related Gene Expression in the Liver

**Figure 5:**
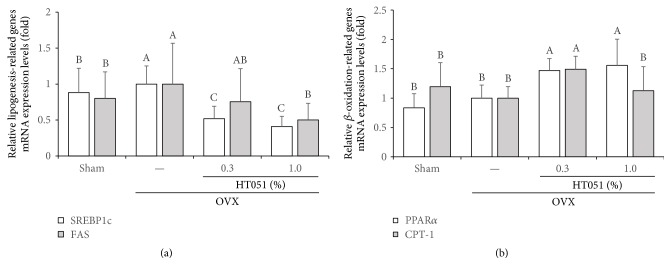
Effects of HT051 on mRNA expression of genes related to hepatic lipogenesis (a) and *β*-oxidation (b) in ovariectomized rats. Each value is the mean ± SD (*n* = 12 per group). Data were analyzed by one-way analysis of variance (ANOVA) followed by Duncan's multiple range test. Values not sharing the same letters are significantly different among the groups at *p* < 0.05.

To confirm the effect of HT051 on lipid metabolism changed by ovariectomy, we measured the transcriptional expression level of lipogenesis- and *β*-oxidation-related genes. As shown in Figure 5(a), expression of lipogenesis-related genes, sterol regulatory element-binding protein 1c (SREBP-1c), and fatty acid synthase (FAS) was significantly lower in the 1.0% HT051 group compared to that in the OVX group (*p* < 0.0014 and *p* < 0.0001, resp.). Furthermore, the expression level of two *β*-oxidation-related genes, carnitine palmitoyl transferase 1 (CPT-1) and peroxisome proliferator-activated receptor *α* (PPAR*α*), was significantly higher in HT051-supplemented groups compared to that in the OVX group (*p* < 0.0001 and *p* < 0.0001, resp., Figure 5(b)). However, there was no significant difference between the Sham and OVX groups regarding *β*-oxidation-related genes.

### 3.6. Adipogenesis- and Inflammation-Related Gene Expression in WAT

**Figure 6:**
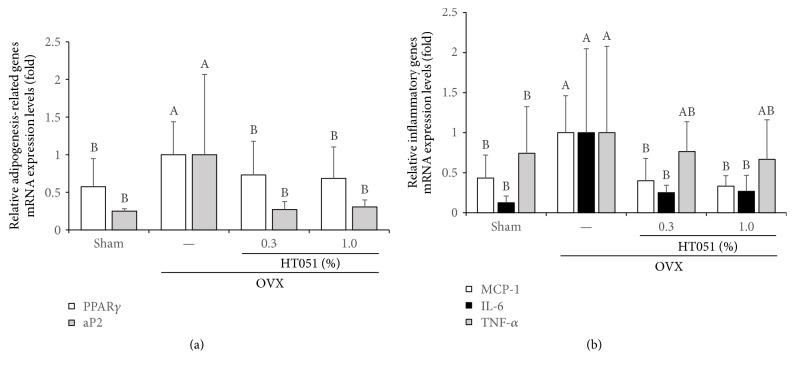
Effects of HT051 on mRNA expression of genes related to WAT adipogenesis (a) and inflammation (b) in ovariectomized rats. Each value is the mean ± SD (*n* = 12 per group). Data were analyzed by one-way analysis of variance (ANOVA) followed by Duncan's multiple range test. Values not sharing the same letters are significantly different among the groups at *p* < 0.05.

To evaluate the effects of HT051 on adipocyte differentiation and fat accumulation in menopause-induced rats by ovariectomy, the mRNA expression level of peroxisome proliferator-activated receptor *γ* (PPAR*γ*) and adipocyte protein 2 (aP2) in WAT was measured (Figure 6(a)). The expression of PPAR*γ* and aP2 mRNA was significantly higher in the OVX group compared with the Sham group (*p* < 0.0001 and *p* < 0.0001, resp.). PPAR*γ* and aP2 mRNA expression was significantly lower in two OVX + HT051 groups compared with the OVX group. In addition, the expression of inflammatory genes including monocyte chemoattractant protein-1 (MCP-1), interleukin-6 (IL-6), and tumor necrosis factor *α* (TNF-*α*) in WAT is shown in Figure 6(b). The OVX group showed the highest levels of expression of WAT inflammatory genes. The level of expression of MCP-1 and IL-6 was significantly lower in two OVX + HT051 groups compared with the OVX group (*p* < 0.0001 and *p* = 0.0083, resp.). The TNF-*α* mRNA expression was lower in the OVX + HT051 group than the OVX group, but there was no significant difference.

## 4. Discussion

In this study, we chose the HT051 supplementation of 0.3–1.0% of the diet. The dose of HT051 consumed by the rats is equivalent to 3.36–11.52 g of* P. lobata* and 0.7–2.4 g of* R. glutinosa* in human dose based on the weight of dried roots, respectively.* P. lobata* and* R. glutinosa* have been used in traditional Korean medicine for a long time and the widely used dose for man in traditional Korean medicine is 12–30 g/day for* P. lobata* and 10–30 g for* R. glutinosa*. Since the dose used in this experiment does not exceed the amount of that traditionally used, the dose is not a problem for human supplementation. HT051 reduced body weight, adipose fat mass, and serum lipid and glucose levels in OVX rats. Moreover, HT051 upregulated the expression of enzymes involved in *β*-oxidation and downregulated the expression of genes involved in lipogenesis, adipogenesis, and inflammation in liver and adipose tissue of OVX rats.

The uterus weight and serum 17*β*-estradiol concentration in the OVX groups decreased compared to those in the Sham group, confirming that ovariectomy successfully induces surgical menopause. Estrogen deprivation in postmenopausal women promotes metabolic syndrome such as obesity, insulin resistance, hyperglycemia, and dyslipidemia [4]. OVX operation results in estrogen deficiency and loss of ovarian function, which is generally accompanied by increased body weight in the form of excessive fat accumulation in human and animals [22]. Actually, OVX model has been used for not only osteoporosis in menopause, but also obesity in menopause [23, 24]. In our experiments, body and body fat weight dramatically increased in OVX rats and significantly decreased in OVX rats fed dietary HT051, without affecting uterus weight or serum estrogen concentration. These results suggest that HT051 might have antiobesity effects in postmenopausal women, without the influence of estrogen.

Dietary HT051 supplementation significantly reduced the serum lipid and glucose in OVX rats. Total body fat weight is strongly associated with serum lipid and glucose concentration, because excess adipocytes alter glucose and lipid homeostasis [25, 26]. Excess fat accumulation releases free fatty acids (FFA), one of the adipose tissue-derived factors, and the enhanced FFA leads to increased serum TG and TC [27]. Moreover, increased FFA could lead to elevation of glucose by stimulating hepatic gluconeogenesis [28]. These results suggest that HT051 supplementation might prevent hyperlipidemia and hyperglycemia in postmenopausal obesity.

Liver weight and serum AST and ALT levels were significantly higher in the OVX group than in the Sham group, and these biomarkers were significantly lower in OVX rats fed with HT051 supplementation compared to that in OVX rats. Estrogen deprivation by ovariectomy in animals leads to increased fat mass and hepatic steatosis [29]. Hepatic steatosis is characterized by increased liver weight and increased serum ALT and AST concentrations [30, 31]. A previous study demonstrated that postmenopausal women have a higher risk of developing NAFLD when compared with premenopausal women because estrogen deprivation causes the accumulation of body fat and hepatic lipid, which can develop and progress NAFLD [32]. Based on our results, HT051 improves hepatic fat accumulation and may help to prevent NAFLD in postmenopausal women.

Dietary HT051 supplementation inhibited the expression of lipogenic genes, including SREBP1c and FAS, and induced the expression of fatty acid oxidation genes, including PPAR*α* and CPT-1, in OVX rats. Hepatic lipid metabolism seems to be regulated by changes in the expression of genes related to lipogenesis and fat *β*-oxidation [33]. SREBP1c, a transcription factor that targets important lipogenic genes, stimulates lipogenesis, the process of fatty acid and TG synthesis, and it increases the expression of lipogenesis-related genes, such as FAS [34]. Fatty acid *β*-oxidation is of multiple catabolic processes by which fatty acids are broken down by the expression of various genes, such as PPAR*α* and CPT-1 [35]. PPAR*α*, in the superfamily of nuclear receptors, upregulates genes involved in cellular fatty acid uptake and transport, such as CPT-1, for fatty acid oxidation [36]. These results demonstrated that HT051 improves postmenopausal obesity by inhibiting lipogenesis and inducing fat oxidation in the liver.

HT051 supplementation significantly suppressed the expression of adipogenic gene PPAR*γ* and aP2 and inflammatory markers, such as IL-6 and MCP-1 in WAT. Adipocytes are the fat cells that primarily compose WAT, and the main function of adipocytes is to regulate energy balance by storing and mobilizing TG. Adipogenesis is the process by which preadipocytes transformed into differentiated adipocytes, and this process is regulated by transcription factor such as PPAR*γ*. PPAR*γ* is highly expressed in adipocytes and is required for stimulating expression of genes related to adipogenesis, such as aP2 [37]. Fatty acid binding protein 4, called aP2, facilitates the shuttling of fatty acids and is primarily expressed in adipocytes and macrophages. It has been proposed to be a marker of adipogenesis because it is highly expressed during adipocyte differentiation [38]. Previous studies have demonstrated that PPAR*γ* and aP2 play an important role in lipid metabolism, insulin resistance, hyperglycemia, and atherosclerosis [39]. Mature white adipocytes secrete various inflammatory cytokines, such as IL-6, TNF-*α*, and MCP-1 [40]. Moreover, estrogen deprivation promotes immune cell infiltration and increased tissue inflammation because estrogen influences immune and inflammatory conditions [41]. Indeed, adipose tissue inflammation is elevated in OVX animals and postmenopausal women [42–44]. It is believed that the increase in proinflammatory cytokines contributes to the development of various chronic diseases, including osteoporosis, atherosclerosis, liver disease, and cancer [27, 45]. Our results demonstrated that decreased fat mass in OVX HT051-fed rats is strongly associated with the downregulation of adipogenic and proinflammatory genes.

## 5. Conclusion

In summary, dietary HT051 supplementation improves postmenopausal obesity by activating fat oxidation and suppressing lipogenesis, adipogenesis, and inflammation in the liver and WAT. Based on these findings, HT051 may be a promising alternative to HRT for the management of postmenopausal obesity.

## Figures and Tables

**Table 1:** Composition of the diets.

	Normal diet	OVX + 0.3% HT051	OVX + 1.0% HT051
Casein	200	200	200
Cornstarch	397.486	397.486	397.486
Dextrin	132	132	132
Sucrose	100	100	100
Cellulose	50	50	50
Corn oil	70	70	70
t-Butylhydroquinone	0.014	0.014	0.014
Salt mix #210025	35	35	35
Vitamin mix #310025	10	10	10
L-Cystein	3	3	3
Choline bitartrate	2.5	2.5	2.5
HT051	0	3	10

Total (g)	1000	1003	1010

**Table 2:** Sequences of primers used for real-time quantitative PCR analysis.

Gene	Forward primer (5′-3′)	Reverse primer (5′-3′)
GAPDH	TGGCCTCCAAGGAGTAAGAAAC	CAGCAACTGAGGGCCTCTCT
SREBP1c	AAAACCAGCCTCCCCAGAGC	CCAGTCCCCATCCACGAAGA′
FAS	GCTAATGCCTACCTGAGTCACAC	GACAACTTAGTCTGCTGCTCTCTG
PPAR*α*	TGGAGTCCACGCATGTGAAG	CGCCAGCTTTAGCCGAATAG
CPT-1	TAGGACAGGCAGAAAATTGC	CAGTAGGAGCCGATTCAAAA
PPAR*γ*	CCCTGGCAAAGCATTTGTAT	GGTGATTTGTCTGTTGTCTTTCC
aP2	GGCTTCGCCACCAGGAA	CCCTTCTACGCTGATGATCAAGT
MCP-1	AATGAGTCGGCTGGAGAACTAC	GATCTCTCTCTTGAGCTTGGTGAC
IL-6	CAGACCTAATGCAGAGAAGTAGCC	GAGCCATCAGTCCTCCATATCTAC
TNF-*α*	GGCAGGTCTACTTTGGAGTCAT	GAGTAGACGATAAAGGGGTCAGAG

**Table 3:** Food intake.

	Sham	OVX	OVX + 0.3% HT051	OVX + 1.0% HT051
Food intake (g/day)	15.49 ± 1.43	16.43 ± 1.11	15.72 ± 1.34	16.25 ± 1.19

**Table 4:** Effects of 8-week administration of HT051 on organ weight in OVX rats.

Weight (g)	Sham	OVX	OVX + 0.3% HT051	OVX + 1.0% HT051
Liver	6.37 ± 0.61^b^	6.98 ± 0.50^a^	6.03 ± 0.71^b^	6.35 ± 0.84^b^
Spleen	0.56 ± 0.12	0.66 ± 0.12	0.64 ± 0.14	0.62 ± 0.10
Total WAT	15.83 ± 4.27^c^	33.17 ± 8.81^a^	26.89 ± 4.62^b^	11.83 ± 2.71^c^
Mesenteric fat	3.70 ± 1.13^c^	9.39 ± 2.76^a^	7.00 ± 2.02^b^	3.54 ± 1.94^c^
Abdominal fat	3.37 ± 1.28^c^	6.58 ± 1.69^a^	4.67 ± 1.25^b^	2.57 ± 0.91^c^
Gonadal fat	4.40 ± 1.76^b^	7.89 ± 2.99^a^	7.14 ± 1.72^a^	2.92 ± 1.12^b^
Perirenal fat	4.36 ± 1.32^b^	9.32 ± 2.37^a^	8.08 ± 2.39^a^	2.79 ± 0.88^b^

Each value is the mean ± SD (*n* = 12 per group) values. Data were analyzed by one-way analysis of variance (ANOVA) followed by Duncan's multiple range test. Values not sharing the same letters are significantly different among the groups at *p* < 0.05.

## Abbreviations

WAT: White adipose tissue
BAT: Brown adipose tissue
NAFLD: Nonalcohol fatty liver disease
VMS: Vasomotor symptoms
HRT: Hormone replacement therapy
OVX: Ovariectomized
RANKL: Nuclear factor kappa-B ligand
FABPpm: Plasma membrane-bound fatty acid binding protein
ANOVA: Analysis of variance
TG: Triglyceride
TC: Total cholesterol
ALT: Alanine transaminase
AST: Aspartate transaminase
SREBP-1c: Sterol regulatory element-binding protein 1c
FAS: Fatty acid synthase
CPT-1: Carnitine palmitoyl transferase 1
PPAR*α*: Peroxisome proliferator-activated receptor *α*
PPAR*γ*: Peroxisome proliferator-activated receptor *γ*
aP2: Adipocyte protein 2
MCP-1: Monocyte chemoattractant protein-1
IL-6: Interleukin-6
TNF-*α*: Tumor necrosis factor *α*
FFA: Free fatty acids.
